# Cancer Moonshot Immuno-Oncology Translational Network (IOTN): accelerating the clinical translation of basic discoveries for improving immunotherapy and immunoprevention of cancer

**DOI:** 10.1136/jitc-2020-000796

**Published:** 2020-06-17

**Authors:** Ananth Annapragada, Andrew Sikora, Catherine Bollard, Jose Conejo-Garcia, Conrad Russell Cruz, Shadmehr Demehri, Michael Demetriou, Levon Demirdjian, Lawrence Fong, Mary Horowitz, Alan Hutson, Kathryn Kadash-Edmondson, Donald Kufe, Steven Lipkin, Song Liu, Claire McCarthy, Martin Morgan, Zachary Morris, Yang Pan, Marcelo Pasquini, Stephen Schoenberger, Eliezer Van Allen, Eduardo Vilar, Yi Xing, Wenjuan Zha, the IOTN Consortium, Adekunle Odunsi

**Affiliations:** 1 Singleton Department of Pediatric Radiology, Texas Children’s Hospital, Houston, Texas, USA; 2 Bobby R. Alford Department of Otolaryngology-Head and Neck Surgery, Baylor College of Medicine, Houston, Texas, USA; 3 Center for Cancer and Immunology Research, Children’s National Medical Center, Washington, District of Columbia, USA; 4 Department of Immunology, H Lee Moffitt Cancer Center and Research Center, Tampa, Florida, USA; 5 Department of Dermatology, Massachusetts General Hospital, Boston, Massachusetts, USA; 6 Department of Neurology, University of California Irvine, Irvine, California, USA; 7 Center for Computational and Genomic Medicine, The Children’s Hospital of Philadelphia, Philadelphia, Pennsylvania, USA; 8 Department of Hematology and Oncology, University of California San Francisco, San Francisco, California, USA; 9 Department of Medicine, Medical College of Wisconsin and Center for International Blood and Marrow Transplant Research, Milwaukee, Wisconsin, USA; 10 Department of Biostatistics and Bioinformatics, Roswell Park Cancer Institute, Buffalo, New York, USA; 11 Department of Medical Oncology, Dana Farber Cancer Institute, Boston, Massachusetts, USA; 12 Department of Medicine, Weill Cornell Medicine, New York, New York, USA; 13 Division of Cancer Biology, National Cancer Institute, Rockville, Maryland, USA; 14 Department of Human Oncology, University of Wisconsin Madison School of Medicine and Public Health, Madison, Wisconsin, USA; 15 Division of Developmental Immunology, La Jolla Institute for Allergy and Immunology, La Jolla, California, USA; 16 Department of Clinical Cancer Prevention, University of Texas MD Anderson Cancer Center, Houston, Texas, USA; 17 Department of Gynecologic Oncology, Roswell Park Cancer Institute, Buffalo, New York, USA

**Keywords:** immunotherapy, immunity

## Abstract

Despite regulatory approval of several immune-based treatments for cancer in the past decade, a number of barriers remain to be addressed in order to fully harness the therapeutic potential of the immune system and provide benefits for patients with cancer. As part of the Cancer Moonshot initiative, the Immuno-Oncology Translational Network (IOTN) was established to accelerate the translation of basic discoveries to improve immunotherapy outcomes across the spectrum of adult cancers and to develop immune-based approaches that prevent cancers before they occur. The IOTN currently consists of 32 academic institutions in the USA. By leveraging cutting-edge preclinical research in immunotherapy and immunoprevention, open data and resource sharing, and fostering highly collaborative team science across the immuno-oncology ecosystem, the IOTN is designed to accelerate the generation of novel mechanism-driven immune-based cancer prevention and therapies, and the development of safe and effective personalized immuno-oncology approaches.

## Introduction

**Figure 1:**
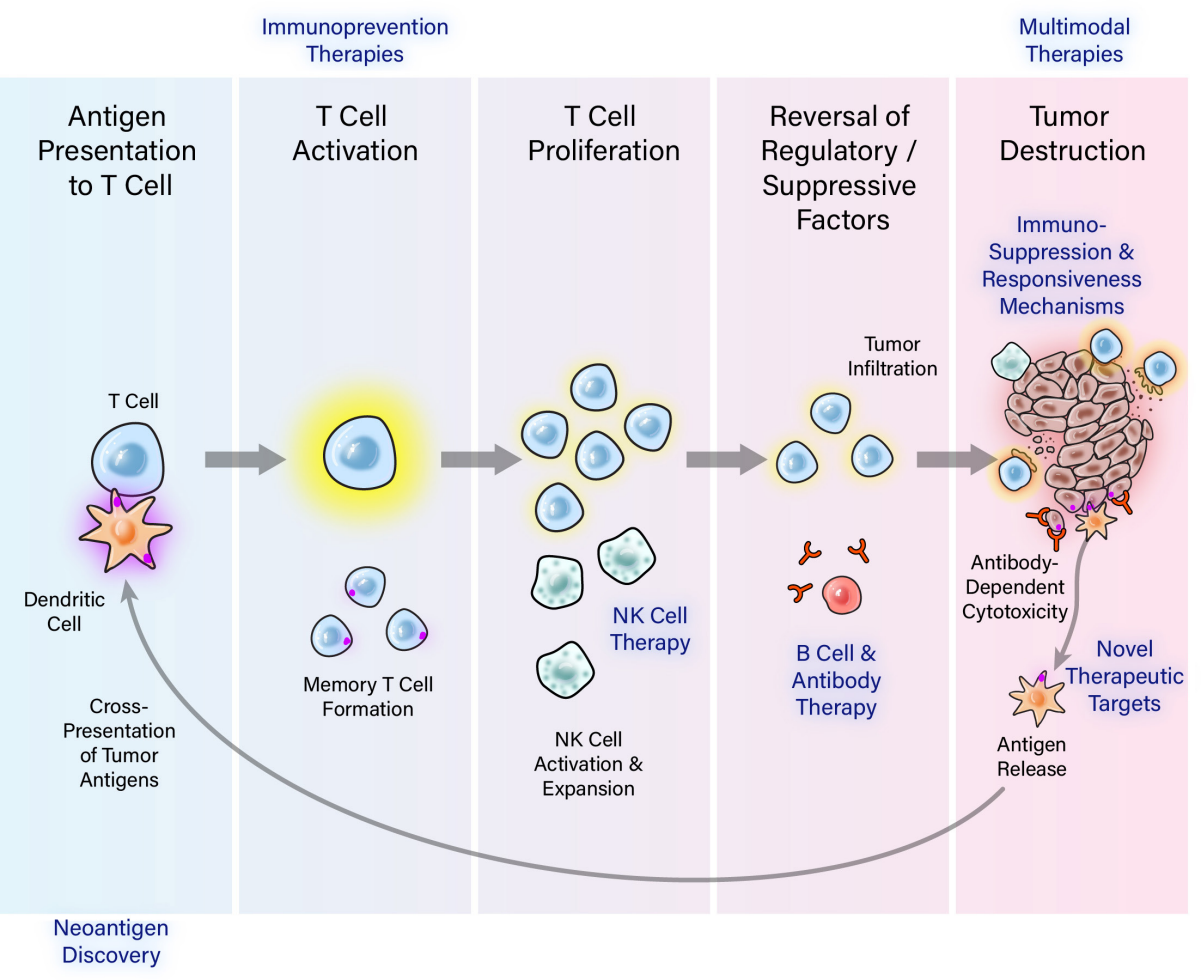
Critical steps of effective antitumor immunity and the Immuno-Oncology Translational Network (IOTN) research projects tackling the steps. The immunological destruction of tumors is a multistep, coordinated process that can be modulated or targeted at several critical points to elicit tumor rejection. These steps include: (1) processing and presentation of a tumor-specific antigen by antigen-presenting cells, (2) generation of sufficient numbers of effector T, B or natural killer (NK) cells in vivo; (3) trafficking and infiltration into the tumor; (4) overcoming inhibitory networks in the tumor microenvironment; and (5) persistence of the antitumor T cells and the generation of memory T cells. The twelve 2018 IOTN awards tackle several key aspects (highlighted in blue) in this process.

Immunotherapy is based on the principle that the immune system can recognize tumor-associated antigens and direct a targeted response (figure 1). While immunotherapy has emerged as the most promising cancer treatment since the development of chemotherapy in the 1940s, the majority of patients with cancer do not yet obtain durable benefit from immunotherapies, thus underscoring the need to understand mechanisms of immune resistance, identify biomarkers of responsiveness and develop novel approaches. The ultimate goal of immuno-oncology is to improve the clinical impact and cost/benefit ratio of immunotherapy by extending immunotherapeutic approaches to more patients and tumor types. To accomplish this goal, immuno-oncology research must address basic, translational and clinical research barriers, thus harnessing the full therapeutic potential of the immune system.

**Figure 2:**
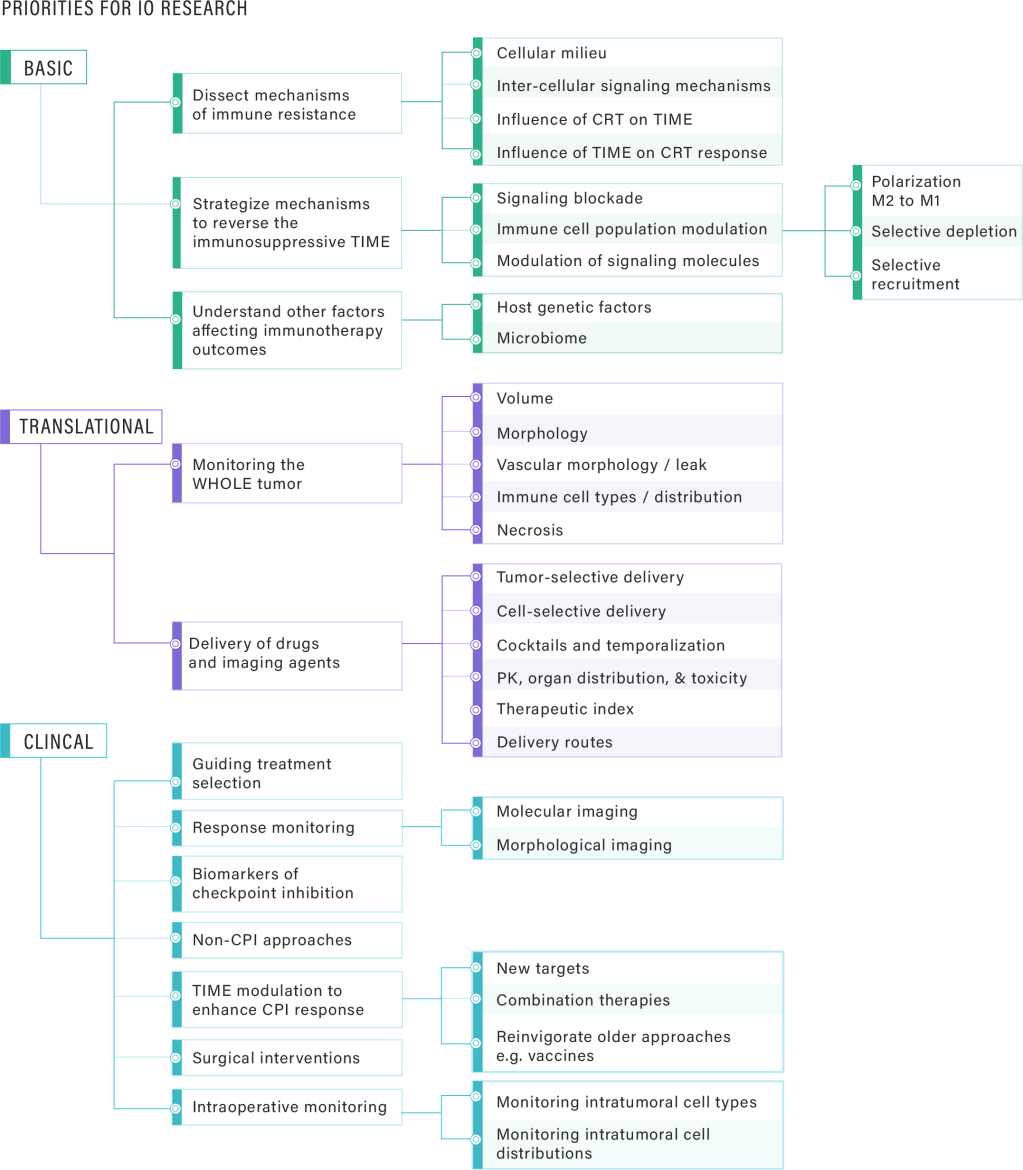
Immuno-oncology (IO) research priorities outlined at the basic, translational and clinical levels. At the basic level, unraveling the fundamental mechanisms of immune resistance is critical, including the interplay between the cells of the tumor immune microenvironment (TIME), conventional treatments, and treatment response. At the translational level, there are critical needs in methods to monitor the “whole” tumor—beyond just size, including characterization of the vascular network, vessel leakiness, necrotic zones, hypoxia and cell types/cell distributions in the TIME. Equally important are methods for selective tumor delivery, intratumoral delivery to specific cell types, the spatial and temporal distributions of individual drugs in a cocktail, and appropriate measures of therapeutic index. At the clinical level, it is crucial to define clinically meaningful endpoints for the combination of surgical intervention with immunotherapeutic approaches, and intraoperative monitoring methods that quantify the tumor as well as the TIME. Interfacing with basic and translational areas, the priorities for clinical research include improving the efficacy and safety profiles of existing agents, leveraging the development of novel targets, platforms and delivery systems for new immune-oncology therapeutics, integration of tools and approaches for patient selection, and monitoring of responses to IO therapy. CRT, chemoradiotherapy; PK, pharmacokinetics; CPI, checkpoint inhibitor.

It is now recognized that cancer cells have evolved numerous strategies for evading antitumor immunity. The diversity of tumor–host interactions highlights (1) real-time evolution of immune resistance under selective pressure of immunotherapy; (2) immune escape mechanisms that prevent endogenous antitumor immune responses and limit response to immunotherapy; (3) the need to simultaneously target multiple immune escape mechanisms, potentially in combination with conventional therapies; and (4) the need for immunotherapy approaches to induce a virtuous cycle of antigen release and cross presentation that continuously magnifies and broadens the immune response. Considering the current landscape of immuno-oncology approaches (eg, cancer vaccines, cell-based therapy, bispecific antibodies, immune checkpoint blockade, and oncolytic virus-based therapy), interrelated research priorities and unmet needs exist at the basic, translational and clinical levels (figure 2). To make advances related to these research priorities, it has become increasingly clear that collaborative and coordinated efforts are needed in immuno-oncology that integrate multidisciplinary approaches and innovative technologies.

## Immuno-Oncology Translational Network

As stated in its 2016 report, the overarching goal of the Cancer Moonshot^SM^ is “to achieve a decade’s worth of progress in preventing, diagnosing, and treating cancer in five years, ultimately striving to end cancer as we know it.”^1^ A Blue Ribbon Panel of experts provided 10 recommendations to accelerate cancer research. One was to “create a translational science network devoted exclusively to immunotherapy,” which led to the formation of the “Immuno-Oncology Translational Network” (IOTN). The scope of the IOTN encompasses preclinical research on the entire spectrum of adult cancers to improve the efficacy, durability, and safety of immunotherapy, and to develop immunoprevention approaches. The collaborative approaches used by the IOTN including the *Steering Committee*, its *Working Groups*, and *two cross-network data sharing resources*, the *Data Management and Resource Sharing Center (DMRC)*, and the *Cellular Immunotherapy Data Resource (CIDR)*, are described in the rest of this document. They are designed to facilitate rapid translation of basic discoveries to translation and clinical application, by IOTN-funded researchers and the broader research community, thus contributing to the Cancer Moonshot goal of a decade’s worth of progress in 5 years.

### IOTN membership and consortium structure

Programmatic stewardship of the IOTN resides in a cross-National Institutes of Health (NIH) adult immunotherapy implementation team, comprised ~30 science and medical officers across 11 NIH institutes and centers. The NIH implementation team provides scientific and programmatic oversight to ensure the IOTN meets the goals defined by the Cancer Moonshot Blue Ribbon Panel.

**Figure 3:**
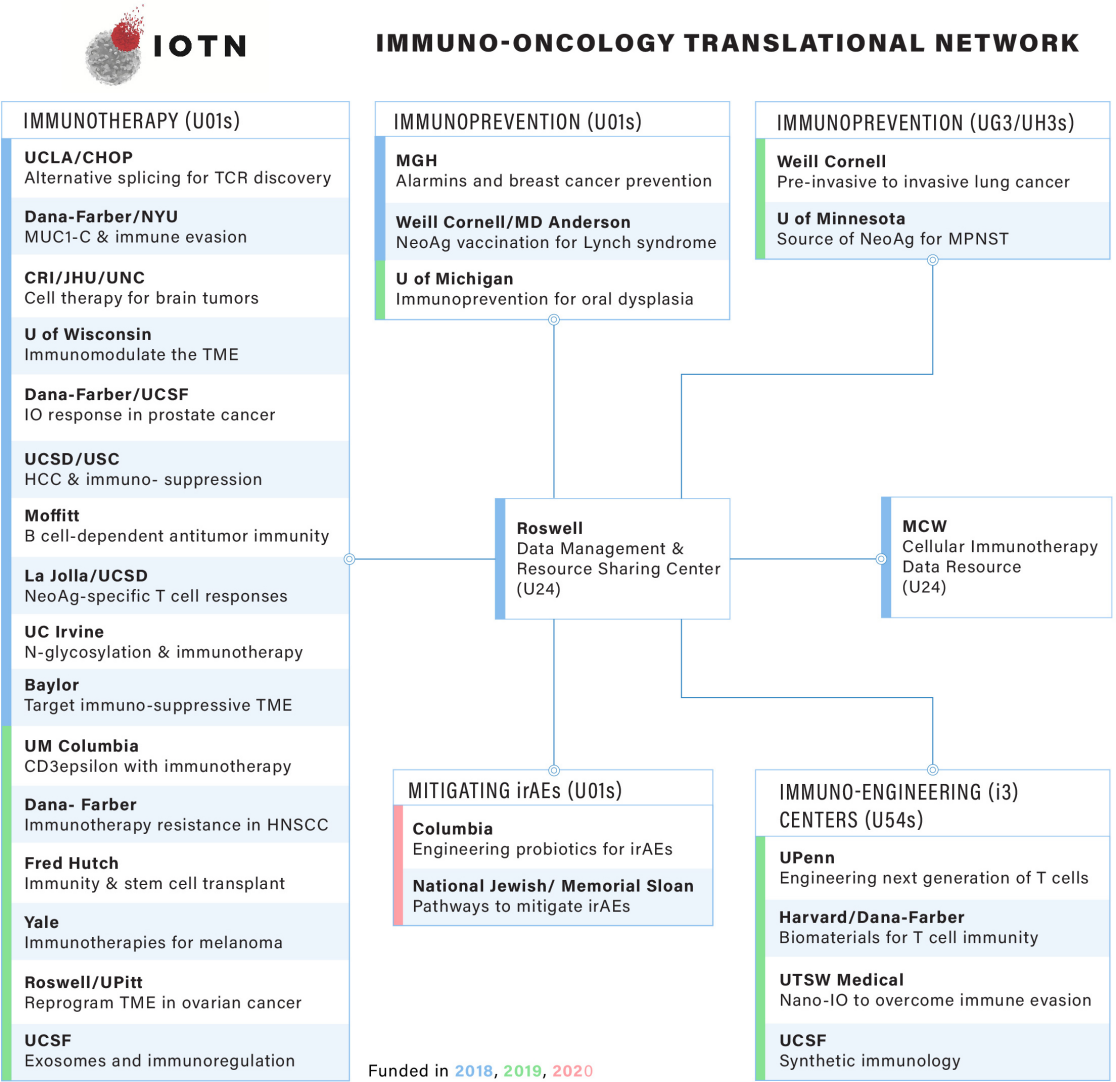
The current IOTN research. The IOTN currently has 16 U01 awards in immunotherapy, 3 U01 and 2 UG3/UH3 awards in immunoprevention, 4 U54 immuno-engineering (i3) centers, 2 U01 awards in immune-related adverse events (irAEs) and 2 U24 coordination centers. HCC, hepatocellular carcinoma; HNSCC, head and neck squamous cell carcinoma; MUC1, oncoprotein mucin 1; TME, tumor microenvironment; MPNST, Malignant Peripheral Nerve Sheath Tumor; UCLA, University of California, Los Angeles; UCSF, University of California, San Francisco; UCSD, University of California, San Diego; NYU, New York University; CRI, Children's Research Institute; JHU, Johns Hopkins University; CHOP, Children's Hospital of Philadelphia; UNC, University of North Carolina; MGH, Massachusetts General Hospital; MCW, Medical College of Wisconsin; UTSW, University of Texas Southwestern; USC, University of Southern California.

**Figure 4:**
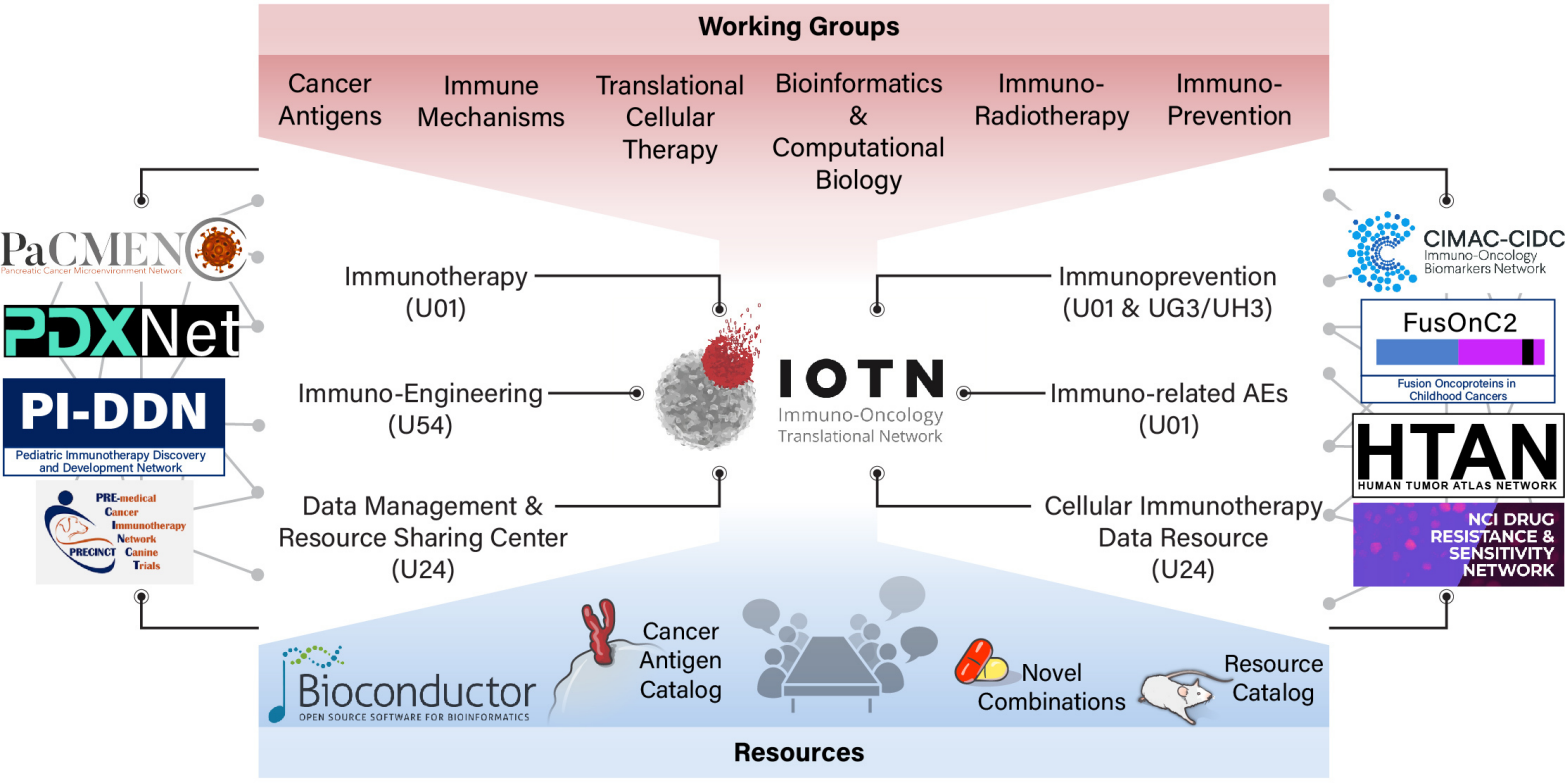
The Immuno-Oncology Translational Network (IOTN) consortium structure and its collaborative networks. The IOTN consortium comprises multiple research areas, working groups and recourses. The IOTN also maintains close interactions with other Moonshot networks, including Pancreatic Cancer Microenvironment Network (PaCMEN), Patient-Derived Xenograft Development and Trials Centers (PDXNet), Pediatric Immunotherapy Discovery and Development Network (PI-DDN), Pre-Medical Cancer Immunotherapy Network for Canine Trials (PRECINCT), Immuno-Oncology Biomarkers Network (CIMAC-CIDC), Fusion Oncoproteins in Childhood Cancers (FusOnC2), Human Tumor Atlas Network (HTAN), and Drug Resistance and Sensitivity Network (DRSN). AEs, adverse events.

The IOTN is governed by a Steering Committee, consisting of representatives of the research investigators and the NIH implementation team. The 2018 inaugural IOTN Steering Committee meeting consisted of a collaborative network of investigators from 14 cooperative agreement awards, spanning 19 institutions (figure 3). At the inception of the IOTN, investigators identified cross-cutting scientific needs and gaps to be addressed by the Steering Committee, and thus formed six working groups, focusing on Cancer Antigens, Immune Mechanisms, Immuno-radiotherapy, Immunoprevention, Translational and Cellular Therapy, and Bioinformatics and Computational Biology (figure 4). These working groups are expected to synergistically address unmet needs beyond the scope of individual projects, thus advancing immuno-oncology research across the IOTN and the larger research community. Throughout these activities, the Steering Committee will seek perspectives, guidance and feedback from patient advocates to ensure meaningful patient engagement. In addition to the Steering Committee, two resource centers, the DMRC and the CIDR provide cross-network support for the IOTN.

### IOTN collaborative networks

Intranetwork and internetwork collaborations are critical aspects of the IOTN. The Pediatric Immunotherapy Discovery and Development Network, a key complementary research initiative of the Cancer Moonshot devoted to advancing immunotherapy for children and adolescents, provides opportunities for collaboration with the IOTN. The IOTN also interacts with other Cancer Moonshot initiatives with scientific interests in immunotherapy through meetings, outreach efforts, collaboration and data sharing activities (figure 4). The Cancer Moonshot Collaborative Meeting in the fall of 2019 brought together the IOTN and eight other Cancer Moonshot networks. Additionally, the IOTN has established an interface with international scientific research organizations such as the Society for Immunotherapy of Cancer and the American Association for Cancer Research.

## Research of the IOTN

The 12 initial IOTN projects funded in 2018 tackle key aspects of the cancer immunity cycle (figure 1) across diverse tumor types, covering immunoprevention, novel therapeutic targets, cell-based therapies, neoantigen and T cell receptor (TCR) approaches, immunosuppression and immunoresponsiveness, and multimodal therapies. In 2019, the IOTN added six new immunotherapy projects utilizing oncolytic viruses, cytokines, exosomes, CD3ε, and hematopoietic stem cell transplantation, as well as three new exploratory immunoprevention projects. In addition, four multidisciplinary immunoengineering centers were established to develop durable, widely accessible, and less toxic adoptive cell therapy strategies. In 2020, two new research projects that seek to reduce the incidence and/or severity of immune-related adverse events will be incorporated to the IOTN portfolio (figure 3 and online supplementary table S1).

10.1136/jitc-2020-000796.supp1 Supplementary data

Each IOTN awardee was selected as top scoring application to the Funding Opportunity Announcements which underwent a competitive review process organized by the National Cancer Institute (online supplementary table S2). The length of U01, U24, and U54 IOTN projects is 5 years. IOTN UG3/UH3 Immunoprevention Projects have a UG3 phase of up to 2 years for exploratory studies and can then transition to a UH3 implementation phase of up to 3 years based on an administrative review of the UG3 milestones.

### Immunoprevention

Since early neoplasms induce less immunosuppression than advanced tumors, the clinical impact of immunoprevention is potentially greater than that of immunotherapy in established tumors. IOTN investigators at Weill Cornell Medical College and The University of Texas MD Anderson seek to delineate recurrent “shared” premalignant neoantigens associated with Lynch syndrome to develop effective and safe vaccination strategies for colorectal cancer^2 3^ and anticipate a clinical trial in 2020. IOTN studies of premalignant lesions at Massachusetts General Hospital enable the discovery of factors critical for initiating antigen presentation and T cell activation, leading to immune elimination of premalignant cells. These efforts have led to the discovery of alarmins, epithelium-derived cytokines with tumor protective effects against early skin and breast carcinogenesis,^4 5^ which have shown cancer immunopreventive efficacy in patients.^6 7^ Ongoing IOTN efforts also target immunoprevention for specific mutation-driven cancers.

### Novel therapeutic targets

To overcome the challenge posed by the dearth of tumor-specific cell surface targets for checkpoint inhibition, bispecific antibody linking of T-cells to cancer cells and chimeric antigen receptor (CAR) T-cell therapy, IOTN investigators at the University of California-Irvine are developing strategies to target cancer-specific glycan antigens known as “tumor associated carbohydrate antigens”^8^ and develop glycan-dependent T cell recruiter technology. In parallel, IOTN researchers at Dana-Farber Cancer Institute are developing agents that target the C-terminal subunit of the oncoprotein mucin 1, which promotes cancer progression and contributes to immune evasion.^9^

### Cell-based therapies

In addition to support by the CIDR, IOTN aims to develop novel cell-based therapies for high-risk cancers. IOTN investigators at Children’s National Medical Center/The George Washington University, Johns Hopkins University, and the University of North Carolina, Chapel Hill are investigating innate immunity through invariant natural killer-T (iNKT) cells and natural killer (NK) cells, for the treatment of glioblastoma multiforme (GBM). They genetically engineer non-tolerized NK and iNKT cells from healthy donors and patients with a CAR targeting B7H3 expressed on GBM cells to promote antitumor immunity. Another team of IOTN researchers at Moffitt Cancer Center is developing an approach to promote combined humoral and T cell responses through orchestration of Tertiary Lymphoid Structures in ovarian cancer.

### Neoantigen and TCR approaches

IOTN researchers at the University of California, Los Angeles and the Children’s Hospital of Philadelphia are exploiting alternative pre-mRNA splice variants as a source for neoantigen discovery in prostate and lung cancer using multiomic analysis. Epitopes derived from alternative splicing will then be prioritized for the development of TCR-engineered T cell therapies. Additionally, IOTN researchers at La Jolla Institute are identifying tumor-associated neoantigens in head and neck squamous cell carcinoma using a bioinformatic and functional analysis platform and seek to understand the key parameters of therapeutic efficacy for autologous neoantigen-specific T cells through integration with patient-derived xenograft models. The IOTN has also engaged with the Immune Epitope Database (www.iedb.org) to define targets of adaptive immune responses^10^ and enable targeted analysis of cancer epitopes (the Cancer Epitope Database and Analysis Resource).

### Multimodal therapies

**Figure 5:**
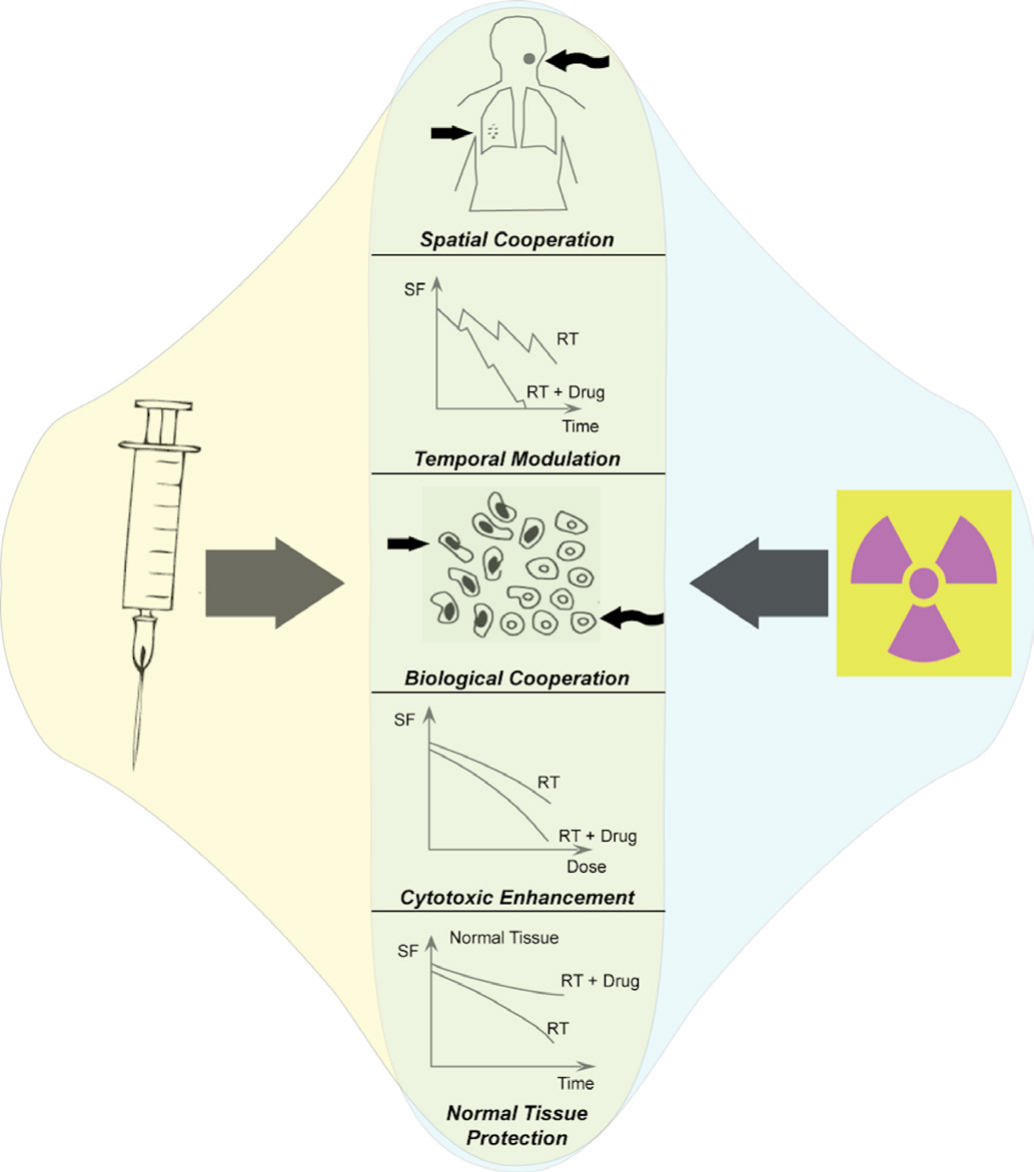
Schematic for conceptualizing potential interactions of immunotherapies and other cancer treatment modalities, using radiotherapy as a representative example. The potentially exploitable interactions of radiation and immune-targeted therapeutics include spatial cooperation, temporal modulation, biological cooperation, cytotoxic enhancement, and normal tissue protection. RT, radiation therapy; SF, surviving fraction of cells.

Immunotherapies can work in combination with other cancer treatments as depicted in figure 5. The IOTN group at Baylor College of Medicine focuses on oral cancer where immune suppressive regulatory T cells and myeloid-derived suppressor cells limit the efficacy of standard-of-care therapies, such as chemotherapy and/or radiation. This group aims to unmask the therapeutic efficacy of chemoradiotherapy combined with checkpoint inhibition through reversal of the highly suppressive tumor immune microenvironment. In a complimentary project, IOTN investigators from the University of Wisconsin are developing novel approaches to propagate antitumor immune responses by combining targeted radionuclide therapies with an in-situ vaccine. This approach utilizes local radiotherapy and intratumoral injection of immunotherapies to convert the targeted tumor site into a nidus for enhanced tumor-specific antigen presentation and adaptive immune recognition.^11 12^

### Immunosuppression and immunoresponsiveness

To understand complex mechanisms to overcome immunosuppression and enhance immunoresponsiveness across cancer types, IOTN investigators at Dana Farber and the University of California, San Francisco are investigating how multiple immune and tumor properties, including tumor-intrinsic epigenetic dysregulation, mediate the immune response in prostate cancer. Further, they are examining how these interactions can be modified through selective targeting. Another IOTN team at the University of California, San Diego and University of Southern California are exploring adaptive immune mechanisms in the development of hepatocellular carcinoma, along with factors that control the outcome of immune checkpoint blockade.

## IOTN coordinating centers: building and sharing of resources

### DMRC: Data Management and Resource-Sharing Center

Leveraging the unique strengths and widespread adoption of Bioconductor,^13 14^ the IOTN DMRC will tackle challenges in the analysis and comprehension of large, heterogeneous, and complex multiomics immuno-oncology data at both single-cell and organism levels. One area of DMRC priority is software innovations to overcome computational memory and throughput constraints. A second priority is the integration of individual researcher experimental results with large scale, uniformly processed data sets generated at single-cell (eg, The Human Cell Atlas) and bulk (eg, The Cancer Genome Atlas, The Human Microbiome Project) levels. The other two areas of DMRC priority are the dissemination of research results in a prompt and Findable, Accessible, Interoperable, Reusable way,^15^ and training programs that enable individual research labs to embrace the nuanced analysis challenges of integrating diverse assay data.

### CIDR: Cellular Immunotherapy Data Resource

The CIDR aims to provide the academic community and pharmaceutical partners with an infrastructure for the collection and use of high-quality patient data, including demographics, disease characteristics, history of anticancer treatment, details of cellular product manufacturing, toxicities and efficacy outcomes. The scope includes any cell-based cancer therapy, including T-cell based adoptive therapy, or genetically modified cells (eg, CAR T-cells). The CIDR data are collected using a web-based electronic data collection system through comprehensive report forms in the cellular therapy registry. So far, the CIDR has captured data from over 1000 recipients of CAR T-cells from 2016 to 2019, including patients who received commercial and non-commercial products. The hallmark of the CIDR is its ability to maximize the use of cell-based therapy data, and to penetrate data silos, making data available more rapidly to multiple users. The vision for the future is a robust data infrastructure, direct data collection from patients using patient reported outcomes or transferred from the Electronic Medical Record, capturing and merging data from multiple sources, and making it available to treatment centers, study sponsors, different stakeholders, the IOTN, and the biomedical community at large in multiple formats, thus maximizing its utilization.

## Conclusions and future directions

As cancer immunotherapy advances, the IOTN is a bold and unique collaborative effort consisting of 32 academic institutions in the USA, with the aim to achieve a decade’s worth of research advancements in 5 years and thereby accelerate progress in the field. Furthermore, the IOTN research centers’ goals of developing molecular signatures that define immune responses within the tumor:immune interface across various cancer types will serve as a rich public resource to address important scientific and clinical questions (table 1). By leveraging cutting-edge preclinical research, and collaborating with stakeholders across the immuno-oncology ecosystem, the IOTN will serve as an engine of translational research into immune-based cancer prevention and therapies. IOTN studies will provide new insights into the dynamics of response to various immunotherapies and find ways to overcome multiple suppressive pathways in the tumor, leading ultimately to the full potential of development of safe and effective personalized immuno-oncology approaches for patients with cancer.

**Table 1:** Public resources in development by the Immuno-Oncology Translational Network (IOTN)

Resource	Description
Data sharing catalog	Inventory of publications and associated primary data from the IOTN awards
Cellular Immunotherapy Data Resource	Data repository of patients treated with cellular immunotherapy
DMRC analytical support	Biostatistics and bioinformatics analytical supports to IO research.
Bioconductor	Open source software for bioinformatics
Antigen catalog	Cancer epitope database and analysis resource
Animal model resource	Animal models for IO preclinical studies
Clinical translation resource	Resources and mechanisms for effective clinical translation of IO research
IO symposia/workshops	Specialty symposia and workshops organized by the IOTN Working Groups

DMRC, Data Management and Resource-Sharing Center; IO, immuno-oncology.
