# Supplementary material for: Cancer Moonshot Immuno-Oncology Translational Network (IOTN): accelerating the clinical translation of basic discoveries for improving immunotherapy and immunoprevention of cancer
Source: J Immunother Cancer. 2020 Jun 17;8(1):e000796. doi: 10.1136/jitc-2020-000796 (PMC7304845; doi:10.1136/jitc-2020-000796)
Supplement: Supplementary data [file jitc-2020-000796supp001.pdf]

Table S1. The current IOTN awards.

Project Number	Project Title	Award Organization	PIs (Lead PI in bold)
UG3CA244697	Intercepting Progression from Pre-invasive to Invasive Lung Adenocarcinoma	Weill Medical College of Cornell University	**N.K. Altorki**, A. Borczuk, O.Elemento, T. Mcgraw, V. Mittal
U01CA233097	Epithelium-Derived Alarmins Role in Breast Cancer Immunoprevention	Massachusetts General Hospital	**S. Demehri**
UG3CA244687	Recurrent Tumor-specific Alternately Processed Transcripts as a Source of Neoantigens for NF1-associated Malignant Peripheral Nerve Sheath Tumor Immunoprevention	University of Minnesota	**D. Largaespada**
U01DE029255	Robust Immuno-prevention Strategies for High-risk Oral Epithelial Dysplasia	University of Michigan	**Y. L. Lei**, J.C. Brenner, N. Neamati
U01CA233056	Neoantigen Vaccination for Lynch Syndrome Immunoprevention	Weill Medical College of Cornell University / MD Anderson Cancer Center	**S. Lipkin**, E. Vilar-Sanchez
U01CA244452	Mechanisms of Exosome Driven Immunoregulation of Cancer Progression	University of California, San Francisco	**R. Blelloch**, L. Fong
U01CA239258	Enhancing Cell Therapy for Brain Tumors	Children's Research Institute / Johns Hopkins University / University of North Carolina at Chapel Hill	**C. Bollard**, C.R. Cruz, R. Jones, B. Savoldo
U01CA233096	Cytokine Immunotherapies for Melanoma	Yale University	**M. Bosenberg**, A. Ring
U01CA232758	B Cell-Dependent Anti-Tumor Immunity in Ovarian Cancer	H. Lee Moffitt Cancer Center & Research Institute	**J. Conejo-Garcia**
U01CA233078	N-Glycosylation and Immunotherapy for Cancer	University of California, Irvine	**M. Demetriou**
U01CA244314	Human CD3epsilon Co-potentiation to Boost Immunotherapy	University of Missouri-Columbia	**D. Gil Pagés**
U01CA244291	Optimizing Myeloma-specific Immunity After Autologous Stem Cell Transplantation	Fred Hutchinson Cancer Research Center	**G. Hill**
U01AA027681	Immunosuppressive Mechanisms Responsible for Development of Non-Viral Liver Cancer and Control of its Response to Immune Checkpoint Inhibitors	University of California, San Diego / University of Southern California	**M. Karin**, A. El-Khoueiry, S. Shalapour, H. Tsukamoto
U01CA233084	MUC1-C is a Target for Reversing Immune Evasion and Resistance to Immunotherapies	Dana-Farber Cancer Institute / New York University	**D. Kufe**, K.K. Wong
U01CA233102	Immunomodulation of the Tumor Microenvironment with Molecular Targeted Radiotherapy to Facilitate an Adaptive Anti-Tumor Immune Response to Combined Modality Immunotherapies	University of Wisconsin – Madison	**Z. Morris**, J. Weichert
U01CA233085	Reprograming the Tumor Microenvironment to Overcome Multiple Primary and Acquired Immune	Roswell Park Comprehensive Cancer	**K. Odunsi**, A. Gambotto, D.B. Kozbor

	Resistance Mechanisms in Ovarian Cancer	Center / University of Pittsburgh	
U01DE028227	Stimulating Neo-Antigen Specific T Cell Responses in Head and Neck Cancers	La Jolla Institute for Allergy and Immunology / University of California, San Diego	**S. Schoenberger**, J.S. Gutkind, A. Rao
U01DE028233	Targeting the Immunosuppressive Tumor Microenvironment to Enhance Efficacy of Radiotherapy and Immuno-Radiotherapy for Oral Cancer	Baylor College of Medicine	**A. Sikora**, A. Annapragada
U01DE029188	Defining Mechanisms of Immunotherapy Resistance in Head and Neck Squamous Cell Carcinomas	Dana-Farber Cancer Institute	**R. Uppaluri**, D. Barbie, R. Haddad
U01CA233100	Molecular and Immune Drivers of Immunotherapy Responsiveness in Prostate Cancer	Dana-Farber Cancer Institute / University of California, San Francisco	**E. Van Allen**, L. Fong
U01CA233074	Targeting Alternative Splicing for TCR Discovery in Small Cell Carcinomas	University of California, Los Angeles / Children's Hospital of Philadelphia	**O. Witte**, G. Crooks, Y. Xing
U54CA244719	Nano-immuno-oncology Approaches to Overcome Tumor Immune Evasion	University of Texas Southwestern Medical Center	**J. Gao**, Z. Chen
U54CA244711	Engineering the Next Generation of T Cells	University of Pennsylvania	**C. June**, G. Linette, M. Milone
U54CA244438	UCSF Center for Synthetic Immunology: Tools to Reprogram the Immune System to Combat Cancer	University of California, San Francisco	**W. Lim**, T. Desai, K. Roybal
U54CA244726	Biomaterials to Create T Cell Immunity	Harvard University / Dana-Farber Cancer Institute	**D. Mooney**, F. Hodi, D. Scadden, W. Shih, C. Wu
U01AR077511	Identification of Pathways to Mitigate Immune-Related Adverse Events with Cancer Immunotherapy	National Jewish Health / Memorial Sloan Kettering Cancer Center	**D.Y. Leung**, J.A. Kern, M. Lacouture
U01CA247573	Engineering Immunotherapeutic Probiotics to Mitigate irAEs	Columbia University	**T. Danino**
U24CA232979	IOTN: Data Management and Resource-Sharing Center	Roswell Park Comprehensive Cancer Center	**A. Hutson**, S. Liu, M. Morgan, K. Odunsi
U24CA233032	IOTN: Cellular Immunotherapy Data Resource (CIDR)	Medical College of Wisconsin	**M. Pasquini**

Table S2. The IOTN initiatives.

FOAs	IOTN Initiative	Length of Initiative	Goal
RFA-CA-17-045/ RFA-CA-19-015	Cancer Immunotherapy Research Projects (U01)	5 years	Improve cancer treatment with immune-based approaches by enhancing our understanding of factors that contribute to immunosuppression, including within the immune cells, tumor cells, or tumor microenvironments.
RFA-CA-17-046/ RFA-CA-19-014	Cancer Immunoprevention Research Projects (U01)	5 years	Identify the earliest exploitable changes in the carcinogenic process and develop preclinical immune-based interventions for prevent the development of cancers.
RFA-CA-17-047	Data Management and Resource-Sharing Center (DMRC) (U24)	5 years	Provide overall support, and facilitate collaboration, data management, and data sharing among the IOTN-funded components, as well as promote scientific outreach with other Cancer Moonshot initiatives and the larger scientific community.
RFA-CA-17-048	Cellular Immunotherapy Data Resource (CIDR) (U24)	5 years	Support a data registry for collecting outcomes of patients receiving cellular immunotherapies, that could be utilized for observational studies or inform subsequent pre-clinical studies and clinical trials.
RFA-CA-19-012	Cancer Immunoprevention Research Projects (UG3/UH3)	UG3 - 2 years<br>UH3 - 3 years	Perform exploratory studies aimed at immune target identification and validation, followed by the development and preclinical testing of specific immunoprevention interventions.
RFA-CA-19-013	Immuno-engineering to Improve Immunotherapy (i3) Centers (U54)	5 years	Incorporate innovative engineering solutions to accelerate the preclinical development of immunotherapeutic and immunopreventive interventions that are more effective, safer, and that could benefit a wider patient population.
RFA-CA-19-044	Advancing Cancer Immunotherapy by Mitigating Immune-Related Adverse Events (irAE) (U01)	5 years	Establish a deeper understanding of the origins and activation pathways leading to inflammatory or autoimmune adverse events that currently limit the use of various immunotherapy regimens in patients.
